# Multi-omics reveals that ST6GAL1 promotes colorectal cancer progression through LGALS3BP sialylation

**DOI:** 10.17305/bb.2025.11663

**Published:** 2025-02-07

**Authors:** Yuanchao Shi, Zhenzhong Pan, Jingwei Duan, Zexing Wang, Yiliang Fang, Bo Tang, Quanlin Guan

**Affiliations:** 1 The First Clinical Academy of Lanzhou University, Lanzhou University, Lanzhou, China; 2 Department of General Surgery and Gastrointestinal Oncology Surgery, Lanzhou University First Hospital, Lanzhou, China; 3 State Key Laboratory of Biotherapy and Cancer Center, West China Hospital, Sichuan University, Chengdu, China; 4 Emergency Department, Peking University Third Hospital, Beijing, China; 5 School of Medicine, Chongqing University, Chongqing, China; 6 Department of Neurology, Army Medical University Xinqiao Hospital, Chongqing, China; 7 Department of General Surgery and Center of Minimal Invasive Gastrointestinal Surgery, Third Military Medical University Southwest Hospital, Chongqing, China

**Keywords:** Colorectal cancer, ST6 β-galactoside α2,6-sialyltransferase, lectin galactoside-binding soluble 3-binding protein, sialylation

## Abstract

ST6 β-galactoside α2,6-sialyltransferase 1 (ST6GAL1), a crucial enzyme for tumor-associated sialic acid modification, has been reported to positively correlate with colorectal cancer (CRC) tumorigenesis; however, the underlying mechanism remains unclear. To elucidate the protumor mechanisms of ST6GAL1, we performed transcriptomic and N-glycoproteomic analyses and *in vitro* assays. We found that *ST6GAL1* was significantly upregulated in tumor samples than in matched normal samples by analyzing fresh clinical samples from public databases (mean mRNA expression level: tumor vs. normal samples ═ 0.002712:0.000966, *P* < 0.05, *n* ═ 22). The in vitro results revealed that *ST6GAL1* overexpression promoted CRC cell proliferation, migration, and chemoresistance, which were significantly blocked by its knockdown. Transcriptomic data showed that many genes related to the four modules (proliferation/cell cycle, migration, motility, and epithelial–mesenchymal transition (EMT) were upregulated after *ST6GAL1* overexpression but downregulated after *ST6GAL1* knockdown. Furthermore, the N-glycoproteomic data revealed that 25 substrates that were sialylated upon *ST6GAL1* overexpression were related to protumor activity. Importantly, we found that knockdown of lectin galactoside-binding soluble 3-binding protein (*LGALS3BP*), a newly identified secreted substrate of ST6GAL1, significantly blocked the proliferation, invasion, and chemoresistance of CRC cells induced by *ST6GAL1* overexpression. Treatment with sialidases (neuraminidases, NAs) also blocked the protumor activity of ST6GAL1. Thus, ST6GAL1-induced increased sialylation of substrates, such as LGALS3BP and upregulation of protumor genes promote CRC tumorigenesis and chemoresistance, which provides important perspectives and new targets for the treatment of CRC.

## Introduction

Colorectal cancer (CRC) is one of the costliest malignant tumors, ranking second in mortality and third in incidence among all cancers [1]. Although significant progress has been made in the diagnosis and treatment of CRC, both incidence and mortality rates continue to rise in low- and middle-income countries [1] and have increased rapidly among younger adults (under 55 years old) since the mid-1990s [2, 3]. Therefore, further exploration of the molecular signatures involved in CRC tumorigenesis is necessary.

Sialylation is a common post-translational modification (PTM) that enhances the diversity of proteins, lipids, and RNA. As a biologically significant form of glycosylation, it plays critical roles in processes, such as embryonic development, neurodevelopment, cellular reprogramming, and immune responses [4]. Abnormal sialylation has been linked to malignant transformation [5], and several sialylated glycoproteins, including prostate-specific antigen (PSA), cancer antigen 125 (CA125), and thyroglobulin, are utilized as clinical cancer biomarkers [6]. Additionally, sialylation modifications contribute to key aspects of tumor progression, including epithelial–mesenchymal transition (EMT) [7, 8], malignant proliferation, migration, invasion [9], anti-apoptosis [10–12], cancer cell stemness [13, 14], and treatment resistance [15, 16]. Despite its importance, relatively few sialic acid-modified molecules have been identified. As a result, the discovery of new sialylation substrates and a deeper understanding of the mechanisms underlying sialylation in tumorigenesis have become areas of growing interest.

Sialic acid is covalently attached to the underlying glycan chain via distinct glycosidic linkages (α2,3, α2,6, or α2,8), catalyzed by sialyltransferases [9]. Abnormal sialylation primarily results from altered sialyltransferase expression. ST6 β-galactoside α2,6-sialyltransferase 1 (ST6GAL1) is the only enzyme capable of synthesizing α2,6-sialylated lactosamine (Sia6LacNAc) by adding α2,6-linked sialic acid to Galβ1,4GlcNAc (lactosamine) chains [17]. Notably, high *ST6GAL1* expression and increased sialylation have been reported in various cancer types, including pancreatic, colorectal, prostate, breast, and ovarian cancers [10, 18]. Furthermore, multiple studies have demonstrated that *ST6GAL1* expression correlates with higher tumor grade, metastasis, and poor prognosis [9, 18]. However, the mechanisms by which ST6GAL1 promotes tumorigenicity remain unclear. ST6GAL1 has been shown to induce EMT in pancreatic cancer cells and mediate chemoresistance through sialylation of the epidermal growth factor receptor [9, 19]. Additionally, sialylation of tumor necrosis factor receptor 1, Fas (CD95), and galectin-3 by ST6GAL1 provides protection against apoptosis in cancer cells [10–12]. Over-sialylation of lysosome-associated membrane protein 1 (LAMP1) leads to increased lysosomal exocytosis of soluble hydrolases and exosomes from tumor cells, thereby promoting cancer cell invasion and metastasis [20]. ST6GAL1 influences cancer progression not only through direct sialylation of substrates but also by modulating other processes beyond PTMs. It promotes TGF-β-dependent EMT in GE11 cells and maintains the mesenchymal state via growth signaling [7]. ST6GAL1 also protects tumor cells against hypoxic stress by enhancing the accumulation of hypoxia-inducible factor 1 subunit α in ovarian and pancreatic cancer cells [21]. Furthermore, *ST6GAL1* upregulation in ovarian and pancreatic carcinomas induces the expression of SRY-box transcription factor 9 (*SOX-9*) and Snail family transcriptional repressor 2 (*SNAI2*), both key tumor-promoting transcription factors [14]. These findings suggest that ST6GAL1 regulates cancer progression through its interactions with signaling networks.

The present study aimed to comprehensively investigate the role of ST6GAL1 and identify key sialylation substrates in CRC. Through transcriptomic and proteomic analyses, we found that four module genes related to tumor progression were upregulated following *ST6GAL1* overexpression. Additionally, we performed modification omics on CRC cell lines to screen for sialic acid–modified substrates and identified galectin-3 binding protein (also known as Mac-2 BP, 90K, or LGALS3BP) as a novel sialylation target. LGALS3BP is highly expressed in tumors and is associated with poor clinical outcomes [22]. However, the detailed regulatory mechanisms underlying this association remain unclear. Therefore, further investigation is needed to elucidate how ST6GAL1 influences CRC progression.

## Materials and methods

### Clinical samples

Twenty-two patients enrolled in the study were admitted to the First Affiliated Hospital of the Army Medical University, PLA, and were pathologically diagnosed with CRC. All patients underwent surgery at this hospital. The clinical samples used in this study were obtained from these patients during their operations. Fresh samples were transported to the laboratory within 30 min. Detailed clinical characteristics are summarized in Supplementary Table 1.

### Cell culture

The human CRC cell lines HCT-116, HT-29, Caco2, SW48, and HEK293T were obtained from the American Type Culture Collection (Rockville, MD, USA). All cell lines were cultured in Dulbecco’s Modified Eagle Medium (DMEM; 6123081; Thermo Fisher Scientific, MA, USA) supplemented with 10% fetal bovine serum (FBS; 2535493P; Invitrogen, MA, USA) and 1% penicillin–streptomycin (2441886; Thermo Fisher Scientific). Cells were maintained in a humidified incubator at 37 ^∘^C with 5% CO_2_. All experiments were conducted using mycoplasma-free cells, which were authenticated through short tandem repeat profiling.

### Cell line construction

To generate cells stably transfected with ST6GAL1, the ST6GAL1 sequence was cloned into the pCDH-CMV-MCS-EF1-copGFP lentiviral vector. HEK293T cells were co-transfected with psPAX2, pMD2.G, and the lentiviral constructs. Supernatants were collected 48 h post-transfection, filtered through 0.45-µm filters, and used to infect target cells in the presence of 2 µg/mL polybrene. GFP-positive cells were subsequently sorted via flow cytometry.

For ST6GAL1 and LGALS3BP knockdown, small hairpin RNAs (shRNAs) targeting ST6GAL1 (CCCAGAAGAGATTCAGCCAAA) or LGALS3BP (GTACTTCTACTCCCGAAGGAT) were cloned into the plko.1-copGFP-PURO or plko.1-Hygro plasmids, respectively. HEK293T cells were transfected with psPAX2, pMD2.G, and the lentiviral constructs. Following viral infection, positive cells were selected with 2 µg/mL puromycin or 100 µg/mL hygromycin B, respectively. ST6GAL1 expression was confirmed by reverse transcription-quantitative polymerase chain reaction (RT-qPCR) and western blotting (WB).

### Flow cytometry

The collected samples were thoroughly chopped and digested with enzymes (1.5 mg/mL collagenase IV [11088866001; Roche, Basel, Switzerland], 1 mg/mL DNase I [10104159001; Roche], and 0.2 mg/mL dispase II [4942078001; Roche]) in basic DMEM at 37 ^∘^C for 30 min. The dissociated cells were then passed through a 70 µm cell strainer and centrifuged. After washing with PBS (10010023; Thermo Fisher Scientific), the cells were resuspended in buffer (PBS supplemented with 1% FBS and 0.1% ethylenediaminetetraacetic acid).

For Sambucus nigra lectin (SNA) staining, the cells were incubated with 20 µg/mL SNA-FITC antibody (FL-1301-2; Vector Laboratories, CA, USA), PE-Cy7 mouse anti-human CD45 antibody (1:200, 557847; BioLegend, CA, USA), and eBioscience™ Fixable Viability Dye eFluor™ 780 (1:400, 65-0865-14; eBioscience, CA, USA), followed by analysis via flow cytometry.

For the detection of tumor cell apoptosis, the cells were incubated with PE-conjugated Annexin V (AV, 1:200; 556422; BD, NJ, USA) in a buffer containing 7-amino-actinomycin D (7-AAD, 1:200; 559925; BD) and analyzed via flow cytometry.

### Colony formation and methyl thiazolyl tetrazolium (MTT) assay

For the colony formation assay, we seeded 250, 500, and 1000 cells per well in a six-well plate, replacing the culture medium after an average of four days. The number of colonies formed was counted after 14 days. For the cell viability assay, we seeded 8000 cells per well in a 96-well plate. Optical density values were measured at various time points using the MTT Cell Proliferation and Cytotoxicity Assay Kit (C009S; Beyotime, Shanghai, China), following the manufacturer’s protocol.

### Scratch assay and cell migration

A total of 1 × 10^6^ cells were incubated overnight in six-well plates, and straight lines were lightly drawn to create scratches. The cells were then cultured in DMEM supplemented with 1% FBS and 2% penicillin–streptomycin for varying durations. Cell migration into the central scratch area was observed at each time point (days 0, 2, and 4).

### Total RNA isolation and quantitative reverse transcription polymerase chain reaction

Total RNA was extracted from tissues using the FastPure Cell/Tissue Total RNA Isolation Kit V2 (RC112-01; Vazyme, Nanjing, China) following the manufacturer’s instructions. The RNA was then reverse transcribed into cDNA using the PrimeScript™ RT Reagent Kit with gDNA Eraser (RR047A; TaKaRa Bio, Shiga, Japan). RT-qPCR was performed with the QuantiTect SYBR Green PCR Kit (1725121; Bio-Rad Laboratories, CA, USA). Primers were designed by Beijing Tsingke Biotech Co., Ltd. and are listed in Supplementary Table 2. Gene expression levels were quantified using the 2^-ΔΔCt^ method, with relative expression levels normalized sequentially to GAPDH and the experimental controls.

### WB

The cell pellet was lysed using radioimmunoprecipitation assay (RIPA) buffer (P0013C; Beyotime) supplemented with phenylmethylsulfonyl fluoride (PMSF; ST507; Beyotime) and phosphatase inhibitors (P1081; Beyotime). Total protein concentration was determined using a bicinchoninic acid (BCA) assay (P0012S; Beyotime). Protein levels were measured using the following antibodies: ST6GAL1 (1:200, AF5924; R&D Systems, MN, USA), hGalectin-3BP (0.2 µg/mL, AF2226; R&D Systems), anti-GAPDH (1:3,000, 2118; Cell Signaling Technology, MA, USA), horseradish peroxidase-conjugated anti-goat secondary antibodies (1:2000, A0208; Beyotime), SNA (1:400, B-2; Vector Laboratories), and SABC (A:B ═ 1:1, 70741; Beyotime). The blots were developed using BeyoECL (P0018S; Beyotime), and densitometry was performed using Fiji (ImageJ; National Institutes of Health).

### Hematoxylin & eosin (HE) and immunohistochemistry (IHC) staining

The samples were fixed in 4% paraformaldehyde for 48 h. Dehydration and paraffin embedding were performed using standard methods. Paraffin-embedded tissue sections were stained with antibodies against ST6GAL1 (AF5924; R&D Systems) or B72.3 (915206; BioLegend). Staining was visualized using the Dako REAL™ EnVision™ Detection System (K5007; Dako, Denmark). The cell nuclei were counterstained with hematoxylin (C0105S; Beyotime) and differentiated with an acid-alcohol fast differentiation solution (C0163S; Beyotime). Additionally, the slides were stained directly with hematoxylin, hydrochloric acid-alcohol, and eosin.

### Immunofluorescence (IFC) staining

Cells were fixed in 1% paraformaldehyde, permeabilized with 0.2% Triton X-100, blocked with 5% BSA, and then incubated with the corresponding antibodies (SNA-FITC, 1:200; Alpha Diagnostic International, TX, USA; SNA15-FITC; SNA-HRP, 1:100; Alpha Diagnostic International; SNA15-HRP; hGalectin-3BP, 0.2 µg/mL; AF2226; R&D Systems). The sections were sealed with an antifade mounting medium supplemented with DAPI (P0131; Beyotime).

### The Cancer Genome Atlas (TCGA) data analysis

Data from TCGA colon adenocarcinoma (COAD) and rectal adenocarcinoma (READ) cohorts were analyzed. *ST6GAL1* expression levels were averaged after the *z*-score normalization of the log-transformed expression profiles. Statistical significance was assessed using Student’s *t*-test. The impact of gene expression on survival was evaluated using a Kaplan–Meier plotter, incorporating RNA-sequencing (RNA-seq) data from TCGA, the European Genome-phenome Archive, and the Gene Expression Omnibus databases for patients with CRC. Survival outcomes were determined based on hazard ratios (HRs) with corresponding 95% confidence intervals and log-rank *P* values.

### Bulk RNA-seq and analysis

Libraries were constructed using the NEBNext Poly(A) mRNA Magnetic Isolation Module Kit (New England Biolabs, MA, USA) and the NEBNext Ultra RNA Library Prep Kit for Illumina paired-end multiplexed sequencing (New England Biolabs). The remaining high-quality reads were aligned to the GRCm38 genome assembly using STAR (version 2.7.10a). Quantification was performed with RSEM, which generated a count table. The resulting matrix was then transformed for downstream analyses. For differential expression and pathway analysis, genes with an adjusted < 0.05 and a log2 fold change > 1 or < −1 were selected.

### Proteomics and glycosylation omics

The samples were lysed using RIPA buffer supplemented with 1% PMSF and 1% phosphatase inhibitor, then sonicated three times on ice with a high-intensity ultrasonic processor (Scientz, China). Protein concentrations were measured using a BCA kit according to the manufacturer’s instructions. The samples were incubated with TCA, washed with precooled acetone, redissolved in tetraethylammonium bromide, ultrasonically dispersed, and digested with trypsin. Subsequently, the samples were reduced with 5-mM dithiothreitol at 37 ^∘^C for 60 min and alkylated with 11-mM iodoacetamide at room temperature for 45 min in the dark. The resulting peptides were desalted using a C18 SPE column. Some protein samples were directly subjected to mass spectrometry and data processing, while others underwent biomaterial-based PTM enrichment for N-glycopeptides. The eluted glycopeptides were desalted using C18 Zip Tips per the manufacturer’s instructions and dried for MS analysis.

### Data analysis of protein and glycosylations via omics

The MS/MS data were processed using the MaxQuant search engine (v.1.6.15.0). To ensure high-quality results, the false discovery rate (FDR) at the spectrum, peptide, and protein levels was set to 1% following further data filtering. Identified proteins included at least one specific peptide, and both peptides and proteins were quantified. Subcellular localization for protein annotation and differential protein proportions was predicted using WolF PSORT software.

Additionally, we integrated bulk RNA-seq results from SW48-empty virus (EV) and SW48-OE samples. For the modification omics data combined with quantitative omics results, we excluded differential expression attributed to deregulated protein expression. N-glycosylation sites were verified using UniProt annotations, while other annotations were predicted based on sequence context using the NetNGlyc algorithm. The ratio of the relative quantitative value of each complete glycopeptide in the sample was used to calculate fold changes. A fold change > 1.5 was considered significantly upregulated, while a fold change < 1/1.5 was considered significantly downregulated for differential glycopeptide screening. Gene Ontology (GO) functional enrichment analysis was performed, with significance assessed using Fisher’s exact test. Cluster analysis, based on the functional enrichment of proteins associated with five different sugar types, was conducted to explore potential associations and functional differences. These analyses included GO terms, Kyoto Encyclopedia of Genes and Genomes (KEGG) pathways, protein domains, Reactome, and WikiPathways annotations. Statistical significance was defined as *P* < 0.05.

### Ethical statement

This study was approved by the Ethical Committee of the First Affiliated Hospital of the Army Medical University, PLA (Approval No. (A) KY2022142), and complied with all relevant ethical regulations. All patients provided written informed consent prior to enrollment and sample collection.

### Statistical analysis

All statistical analyses were conducted using GraphPad Prism version 9 (GraphPad Software, USA) and SPSS version 25.0 (IBM Corp., Armonk, NY, USA) for Windows (Microsoft Corp., Redmond, WA, USA). The normality of continuous variables was assessed using the Shapiro–Wilk test in combination with visual inspection, confirming that all continuous variables followed a normal distribution. Continuous variables were analyzed using Student’s *t*-test and are presented as mean ± standard deviation (SD). Pearson’s correlation coefficient was used to evaluate the association between *ST6GAL1* mRNA and KI-67, while Spearman’s rank correlation was applied to assess the relationship between *ST6GAL1* mRNA and clinical stage in patients. A *P* value of < 0.05 was considered statistically significant.

**Figure 1.**
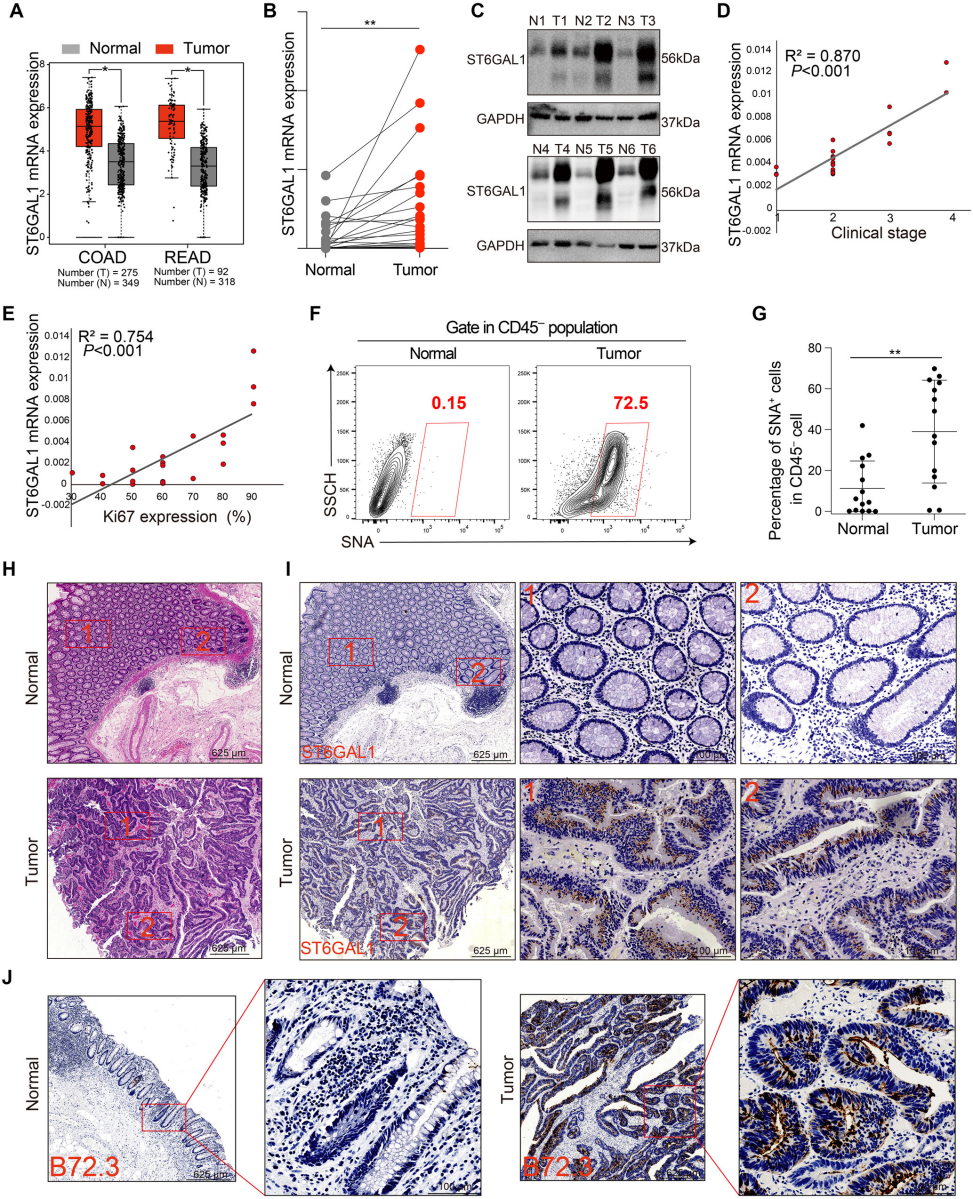
**ST6GAL1 expression and sialylation are strongly increased in the tumor tissue of CRC patients.** (A) ST6GAL1 mRNA expression data were collected from COAD and READ patients in the TCGA database. The corresponding normal tissues in the GTEx database were included as controls. The box plot data was supplied; (B) ST6GAL1 mRNA expression was quantified by reverse transcription-quantitative polymerase chain reaction (RT-qPCR) in fresh tumor tissues compared with normal tissues from 22 clinical CRC patients; (C) The protein level of ST6GAL1 was detected in six clinical samples from CRC patients by WB; (D) Correlation of the ST6GAL1 mRNA level and clinical grade (*n* ═ 22, Spearman correlation coefficient); (E) Correlation between the ST6GAL1 mRNA level and the Ki-67 level (*n* ═ 22; Pearson correlation coefficient); (F) The sialylation level was measured by SNA staining and detected in fresh tumor tissues compared with normal tissue from CRC patients by flow cytometry (*n* ═ 14); (G) Statistical results of the percentage of SNA-positive tumor cells from Figure F; (H and I) Representative HE staining and IHC staining of ST6GAL1 in tumor tissue and adjacent normal tissue from CRC patients (scale bars, 625 µm; scale bars, 100 µm); (J) Representative HE staining and IHC staining of B72.3 in tumor tissue and adjacent normal tissue from CRC patient (scale bars, 625 µm; scale bars, 100 µm). *P < 0.05, **P < 0.01, by Student’s *t*-test (A, B, and G). The data are presented as the mean ± SD of three independent experiments. COAD: Colon adenocarcinoma; READ: Rectum adenocarcinoma; WB: Western blot; SNA: Sambucus nigra lectin; HE: Hematoxylin & eosin; IHC: Immunohistochemistry; TCGA: The Cancer Genome Atlas; CRC: Colorectal cancer.

## Results

### *ST6GAL1* upregulation positively correlates with the clinical stage of CRC

We investigated *ST6GAL1* expression levels and their correlation with α-2,6-sialylation in CRC using TCGA data. Our analysis revealed that *ST6GAL1* expression was significantly higher in CRC tissues compared to normal tissues (Figure 1A). To validate these findings, we analyzed 22 tumor samples and 22 paired adjacent normal samples from CRC patients (clinical information provided in Supplementary Table 1). Although expression levels varied across both groups, *ST6GAL1* transcription was upregulated in 20 out of 22 tumor samples compared to their adjacent normal counterparts (Figure 1B). Similarly, ST6GAL1 protein expression was elevated in tumor samples (Figure 1C). We further examined the correlation between *ST6GAL1* transcription levels, clinical stage, and KI-67 expression. *ST6GAL1* expression in tumor tissues showed a positive correlation with a clinical stage based on diagnostic data (Figure 1D) and with KI-67 expression as determined by IHC analysis (Figure 1E). As ST6GAL1 is the predominant enzyme responsible for adding α-2,6-linkages of sialic acid to N-glycosylated proteins [17], we measured total sialylation levels and observed increased sialylation in tumor samples (Figure 1F and 1G), mirroring the elevated ST6GAL1 protein expression. Additionally, HE and IHC analyses revealed higher *ST6GAL1* expression in tumor regions compared to adjacent normal tissue (Figure 1H and 1I). Since B72.3 recognizes TAG-72, which often carries sialyl-Tn epitopes, we assessed B72.3 expression as an indicator of tumor-associated sialic acid antigens [23]. B72.3 expression was significantly higher in tumor samples (Figure 1J). Collectively, these data indicate that *ST6GAL1* expression and α-2,6-sialylation are significantly upregulated in CRC.

### ST6GAL1 promotes CRC cell tumorigenesis and chemoresistance

**Figure 2.**
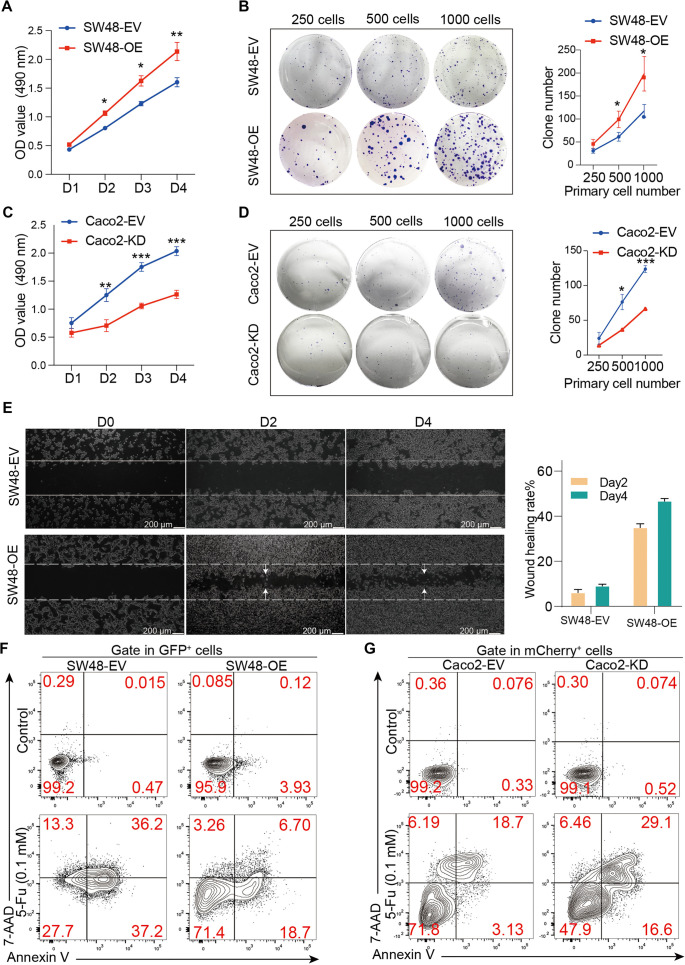
**ST6GAL1 is associated with CRC cell proliferation, migration, and chemoresistance.** After constructing the ST6GAL1-overexpressing (OE) SW48 cell line (SW48-OE) and knockdown (KD) Caco2 cell line (Caco2-KD), as well as the related EV-expressing control cell lines SW48-EV and Caco2-EV, tumor cell viability, colony formation and wound healing were tested. (A and C) Tumor cell viability was measured via the MTT assay in SW48 (A) and Caco2 (C) cells; (B and D) Tumor cell colony formation was detected after 14 days of culture, the numbers of clones were counted, and the statistical results are shown; (E) Wound healing assay of the SW48-EV and SW48-OE cell lines on days 0, 2 and 4 (scale bars, 200 µm) and corresponding statistical results; (F and G) Tumor cells were treated with 5-Fu (0.1 mM) or without 5-Fu for 48 h, and Annexin V and 7-AAD staining was detected by flow cytometry in SW48-EV, SW48-OE, Caco2-EV, and Caco2-KD cell lines. *P < 0.05, **P < 0.01, ***P < 0.001 by Student’s *t*-test (A–D). The data are presented as the mean ± SD of three independent experiments. MTT: Methyl thiazolyl tetrazolium; EV: Empty virus.

To investigate the effect of *ST6GAL1* expression on CRC tumor cells, we employed genetic approaches to establish *ST6GAL1*-overexpressing (*ST6GAL1*-OE) and *ST6GAL1*-knockdown (*ST6GAL1*-KD) tumor cell lines. Baseline expression analysis revealed that the Caco2 cell line exhibited high endogenous *ST6GAL1* expression, whereas the SW48 cell line lacked it (Figure S1A and S1B). Accordingly, *ST6GAL1* was overexpressed in SW48 cells and knocked down in Caco2 cells (Figure S1C and S1D). As expected, confocal microscopy showed that SW48 cells lacked endogenous sialylation, which was markedly increased following *ST6GAL1* overexpression (Figure S1E). Conversely, *ST6GAL1* knockdown in Caco2 cells led to reduced sialylation levels (Figure S1F). These findings were consistent with the WB results (Figure S1G). Interestingly, *ST6GAL1* overexpression in SW48 cells significantly enhanced cell proliferation and colony formation (Figure 2A and 2B), while *ST6GAL1* knockdown in Caco2 cells resulted in decreased proliferation and colony formation (Figure 2C and 2D). Moreover, wound healing was significantly improved by *ST6GAL1* overexpression in SW48 cells, whereas *ST6GAL1* knockdown in Caco2 cells caused a modest but significant reduction in wound healing (Figure 2E). Additionally, following treatment with 5-FU, fewer AV+ and 7-AAD+ cells were detected in the SW48-OE group compared to the SW48-EV group (Figure 2F). In contrast, the Caco2-KD group exhibited an increase in AV+7-AAD+ cells relative to the Caco2-EV group after 5-FU treatment (Figure 2G). Collectively, these data confirm that high *ST6GAL1* expression promotes CRC progression and confers resistance to chemotherapy.

### ST6GAL1 globally enhances the expression of protumor-associated genes

**Figure 3.**
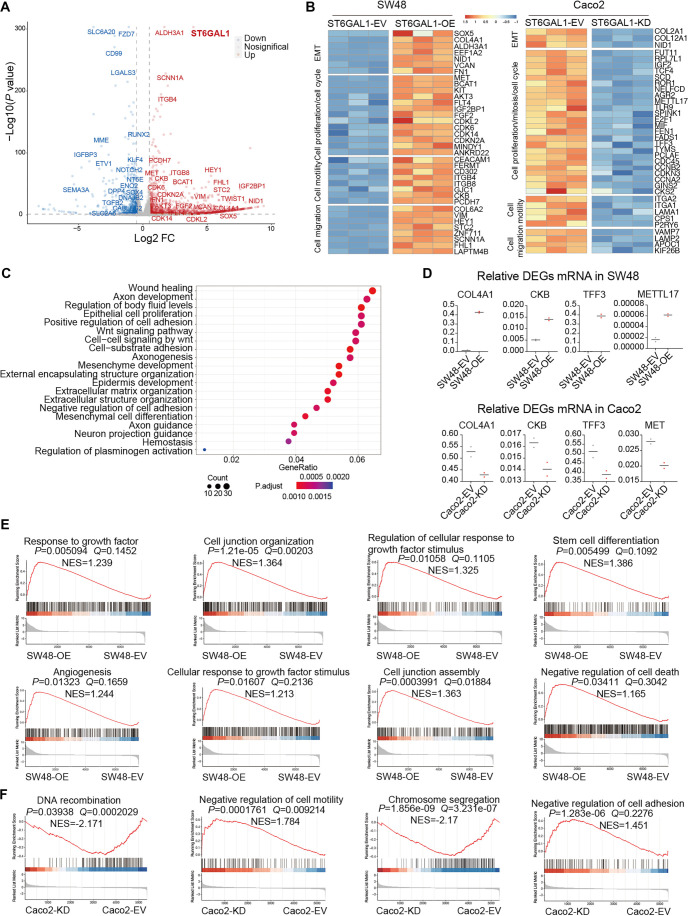
**ST6GAL1 expression mediates transcriptome changes in CRC cells.** Two cell line groups, SW48-EV vs. SW48-OE and Caco2-EV vs. Caco2-KD, were subjected to RNA-seq analysis. (A) Volcano plot showing all genes expressed in the SW48-EV and SW48-OE cell lines. The x-axis shows the log_2_ fold change (FC, SW48-OE vs. SW48-EV), and the y-axis shows the −log_10_ *P* value, which represents the threshold values in log transformation. Each dot represents a differentially expressed gene (DEG). The red dots indicate significantly upregulated DEGs, the blue dots indicate significantly downregulated DEGs, and the gray dots represent nonsignificant DEGs; (B) Heatmap showing the relative expression of selected genes across ST6GAL1-OEand ST6GAL1-KD cell lines in four different functional modules; (C) The transcription levels of some DEGs were confirmed in ST6GAL1-OE and ST6GAL1-KD cell lines via RT-qPCR; (D) GO enrichment analysis of the SW48-EV vs. SW48-OE cell lines; (E) GSEA of the SW48-OE cell line revealed an upregulated signaling pathway compared with that of the SW48-EV cell line. NES, normalized enrichment score; (F) GSEA of the Caco2-KD cell line revealed upregulated and downregulated signaling pathways compared with those in the Caco2-EV cell line. The data are presented as the mean ± SD of three independent experiments (C). DEGs that had *P* < 0.05 and log_2_ FC>1 or <−1 (D, B, E, and F) were selected. RNA-seq: RNA-sequencing; GSEA: Gene set enrichment analysis; GO: Gene Ontology; DEGs: Differentially expressed genes.

To systematically investigate transcriptomic programs related to *ST6GAL1* and CRC progression, we performed RNA-seq on bulk tumor cells from SW48-EV vs. SW48-OE and Caco2-EV vs. Caco2-KD groups (original data shown in Supplementary Tables 3 and 4). In the SW48-EV vs. SW48-OE comparison, 3354 genes were upregulated and 3088 were downregulated (Figure S2A), while 2214 genes were upregulated and 1982 were downregulated in the Caco2-EV vs. Caco2-KD comparison (Figure S2A). RNA-seq confirmed a 16-fold upregulation of *ST6GAL1* in SW48-OE cells (Figure 3A) and a 4-fold downregulation in Caco2-KD cells (Figure S2B). A volcano plot revealed significant upregulation of cell proliferation-related genes, such as *IGF2BP1* (insulin-like growth factor 2 mRNA-binding protein 1) and *BCAT1* (branched-chain amino acid transaminase 1), in the SW48-OE group compared to SW48-EV (Figure 3A). Similarly, cell cycle-related genes, including *CDKN2A* (cyclin-dependent kinase inhibitor 2A) and *CDKL2* (cyclin-dependent kinase-like 2), were upregulated following *ST6GAL1* overexpression (Figure 3A). Additionally, many genes associated with cell migration and motility were significantly upregulated in SW48-OE cells (Figure 3A).

A heatmap analysis showed the global upregulation of genes in four key modules related to proliferation/cell cycle, migration, motility, and EMT (Figure 3B and Supplementary Table 3). Conversely, many genes within these modules were significantly downregulated in Caco2-KD cells compared to Caco2-EV (Figure 3B and Supplementary Table 4). We further validated the expression levels of these genes and found that tumor-promoting genes were upregulated after *ST6GAL1* overexpression in SW48 cells but downregulated following *ST6GAL1* knockdown in Caco2 cells (Figure 3C and Figure S2C and S2D). These findings suggest that *ST6GAL1* promotes tumor growth by regulating pathways involved in proliferation, migration, EMT, and other signaling processes.

Supporting this, GO analysis indicated that *ST6GAL1* overexpression in SW48 cells was positively associated with tumor cell wound healing, proliferation, adhesion, and mesenchymal cell differentiation (Figure 3D). Furthermore, gene set enrichment analysis (GSEA) identified regulatory gene sets linked to growth factor response, cell junction adhesion/assembly, and stem cell differentiation in *ST6GAL1*-overexpressing SW48 cells (Figure 3E and Figure S2E). Pathways related to angiogenesis, negative regulation of cell death, and cell surface receptor signaling were also enriched in SW48-OE cells (Figure 3E and Figure S2E). In contrast, *ST6GAL1* knockdown in Caco2 cells enriched gene sets related to negative regulation of cell adhesion and motility, as well as other pathways associated with reduced cell proliferation (Figure 3F and Figure S2F). Collectively, these data suggest that *ST6GAL1* transcriptionally reprograms tumor cells to enhance proliferation and migration.

### The multiomics landscape of N-glycosylation reveals the protumor activity of sialylation in CRC cells after *ST6GAL1* overexpression

Previous studies have shown that ST6GAL1-induced protein sialylation is involved in tumor cell proliferation, chemoresistance, and tumorigenesis [24]. However, a comprehensive, system-wide view of ST6GAL1-induced sialylation in CRC cells is lacking. To address this, we employed an unbiased N-glycoproteomic approach in *ST6GAL1*-overexpressing SW48 cells. The original quantitative proteomics and N-glycopeptide modification omics data are presented in Supplementary Tables 5 and 6. Overall, we identified 2316 intact glycopeptides (IGPs), mapping to 687 unique N-glycosites across 398 glycoproteins (Figure S3A and S3B; Supplementary Table 6). While the majority of unique N-glycosites (79.48% [546/687]) were annotated as glycosites in the UniProt database (Figure S3B), fewer than half have been reported in the literature. Of these, at least 200 were assigned through “sequence analysis” (Figure S3C). Although most glycoproteins were extracellular (45.83%) or plasma membrane-associated (26.04%), nearly 30.00% were intracellular (Figure S3D). These findings highlight that many N-glycosites, particularly those on intracellular proteins, and their functions warrant further investigation. Approximately 63.03% (433/687) of glycosites were modified by more than one glycan (Figure S3E), and around 32.41% (129/398) of the glycoproteins contained multiple glycosites available for modification (Figure S3F). A heatmap of glycan co-occurrence revealed that pairs among five glycan types—paucimannose, high-mannose, complex/hybrid, fucosylated, and sialylated—frequently co-occurred at the same site, indicating site-specific microheterogeneity and functional complexity (Figure S3G). Compared to the SW48-EV group, the SW48-OE group exhibited increases in 218 IGPs, 87 glycosylated proteins, and 134 glycosylated sites (Figure S4A).

**Figure 4.**
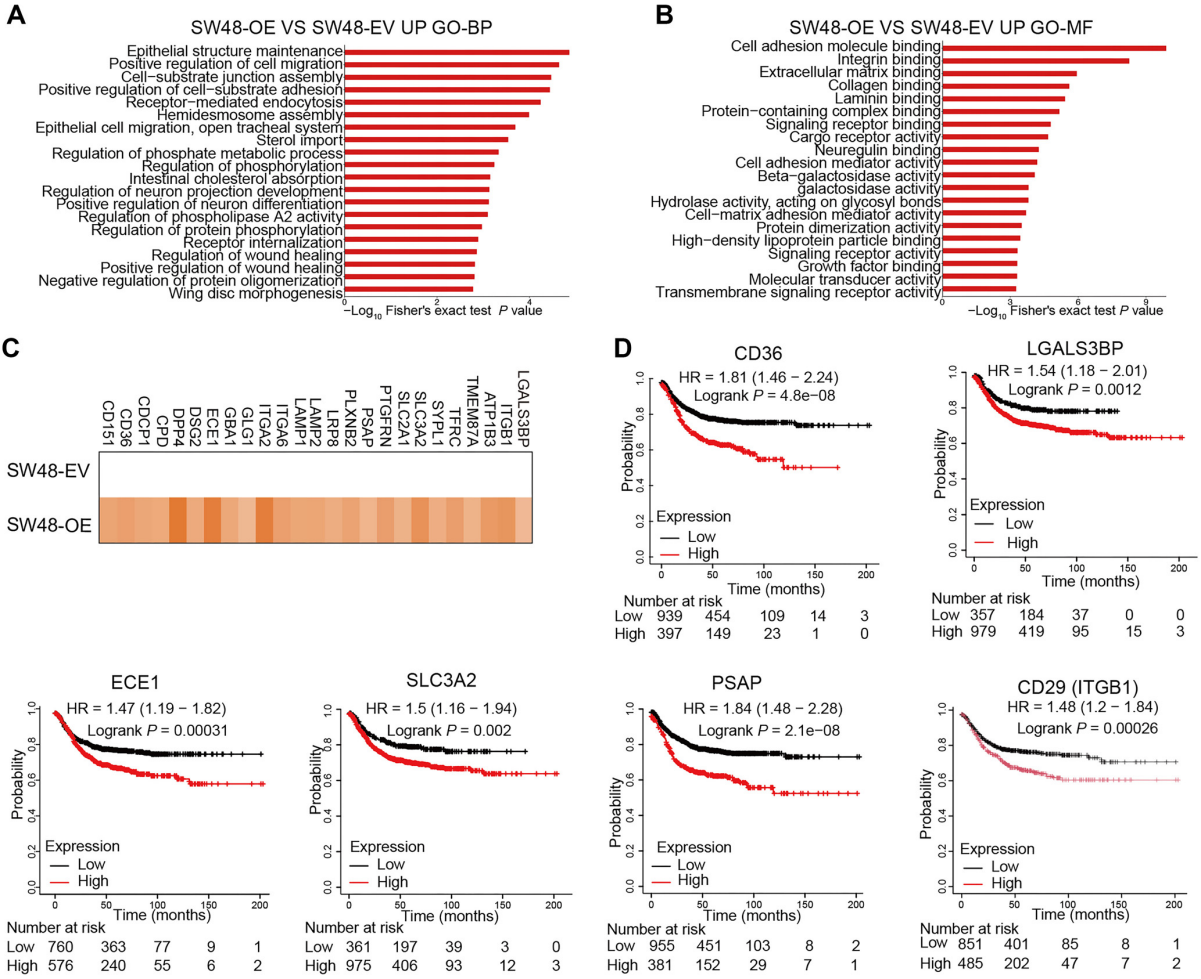
**ST6GAL1 increases tumor cell sialylation, and many sialylated substrates are correlated with tumor progression.** Modification omics was performed on SW48-EV and SW48-OE cell lines, and sialylation was analyzed. (A) Bar plot of BP terms based on the GO enrichment results for sialylation-modified molecules after ST6GAL1 was overexpressed in the SW48 cell line (Fisher’s exact test, *P* < 0.05); (B) Bar plot of molecular function based on GO enrichment results of sialylation-modified molecules after ST6GAL1 was overexpressed in the SW48 cell line (Fisher’s exact test, *P* < 0.05); (C) Heatmap of increased sialylation of proteins in SW48-OE cells compared with SW48-EV cells; (D) Survival analysis of molecules with increased sialylation modifications and Kaplan–Meier plotter database analysis using RNA-seq data from the TCGA, EGA, and GEO databases. BPs: Biological processes; TCGA: The Cancer Genome Atlas; RNA-seq: RNA-sequencing; GO: Gene Ontology.

To gain a global perspective on the effects of *ST6GAL1* overexpression, we performed an unbiased interrogation of GO-based enrichment terms. Ingenuity Pathway Analysis of biological processes (BPs) revealed significant enrichment of GO terms related to “sterol import” and “intestinal cholesterol absorption” in fucosylated samples; “histone lysine methylation” and “negative regulation of protein oligomerization” in paucimannose samples; and “receptor-mediated endocytosis” and the “ubiquitin-dependent ERAD pathway” in high-mannose samples (Figure S4B). However, these findings did not fully explain the observed phenomena. Due to the absence of sialylation data in proteomics databases, we isolated the sialylated proteins for enrichment analysis. This analysis revealed that sialylation was strongly associated with tumor cell migration, wound healing, and adhesion (Figure 4A). Further enrichment analysis highlighted GO terms related to the “regulation of phosphorylation,” which may influence receptor activity, including “growth factor activity” and “signaling receptor activity” (Figure 4B).

We visualized the significantly upregulated sialylated proteins using a heatmap, comparing the SW48-EV and SW48-OE groups (Figure 4C). The observed changes in protein sialylation confirmed the role of ST6GAL1 in sialylation and demonstrated its widespread impact on cellular functions. Consistent with our findings, the omics data identified LAMP1 as a substrate of ST6GAL1 (Figure 4C), and α-2,6 sialylation of LAMP1 has been previously shown to promote cancer cell invasion and metastasis [25]. Sialylation of other proteins, such as SLC3A2, solute carrier family 2 member 1 (commonly referred to as SLC2A1), and ITGB1, has also been reported [26, 27]. Additionally, we identified novel ST6GAL1 substrates, including cell adhesion proteins (LGALS3BP, ATPase Na+/K+ transporting subunit β-3, and transmembrane protein 87A) and ferroptosis-related proteins. Analyzing TCGA data, we found that elevated expression of these proteins was associated with poorer prognosis in CRC patients (Figure 4D and Figure S4C). Taken together, these data suggest that ST6GAL1-mediated sialylation in CRC cells promotes chemoresistance and tumorigenesis.

### LGALS3BP sialylation promotes the malignant progression of *ST6GAL1*-overexpressing CRC cells

**Figure 5.**
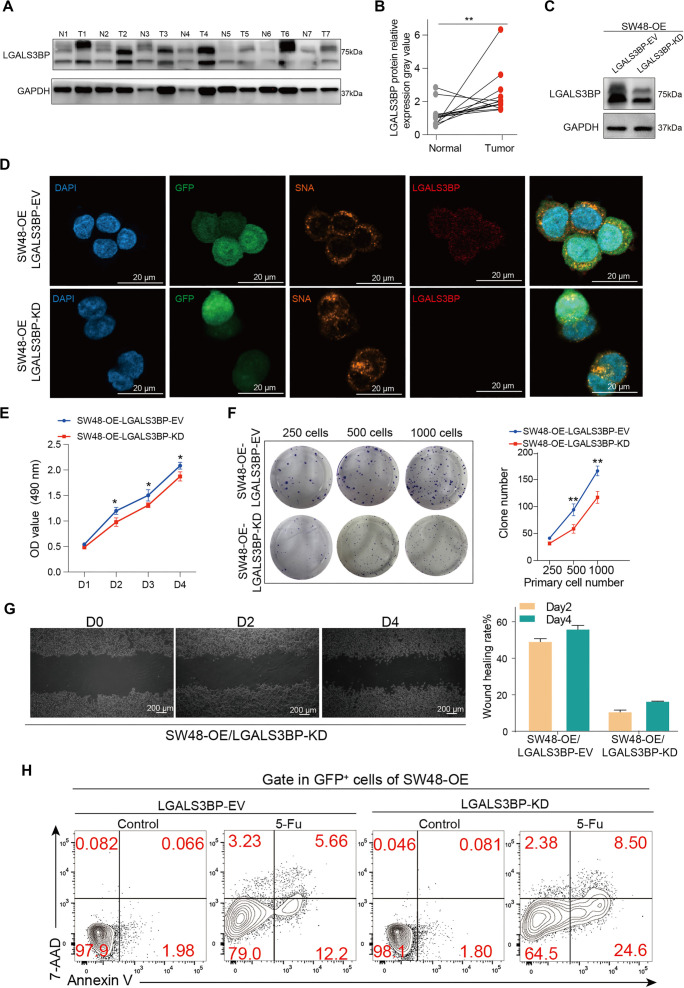
**Sialylation of LGALS3BP promotes the migration of ST6GAL1-OE cells.** (A) WB analysis of LGALS3BP protein levels in tumor and paired normal tissues from CRC patients (*n* ═ 7); (B) Gray value statistical results for CRC patients (*n* ═ 13); (C) LGALS3BP protein expression in LGALS3BP-EV and LGALS3BP-KD cell lines was quantified by WB; (D) Staining of SNA (orange) and LGALS3BP (red) in the LGALS3BP-EV and LGALS3BP-KD of SW48-OE (GFP, green) cell line by IFC. Nuclei were counterstained with DAPI (blue) (scale bars, 20 µm); (E) Tumor cell viability was measured using an MTT assay in LGALS3BP-KD cells; (F) Tumor cell colony formation was detected after 14 days of culture, the number of clones was counted, and the statistical results are shown; (G) Wound healing assay of LGALS3BP-KD in SW48-OE cell line on days 0, 2, and 4 (scale bars, 20 µm) and corresponding statistical results; (H) Tumor cells were treated with or without 5-Fu (0.1 mM) for 48 h, and Annexin V and 7-AAD staining was detected by flow cytometry in LGALS3BP-EV and LGALS3BP-KD of SW48-OE cell line. *P < 0.05, **P < 0.01, by Student’s *t*-test (B, E, and F). The data are presented as the mean ± SD of three independent experiments. IFC: Immunofluorescence; SNA: Sambucus nigra lectin; MTT: Methyl thiazolyl tetrazolium.

LGALS3BP is implicated in the growth and progression of various cancer types, with its protumor activity primarily associated with its secreted form, which depends on N-glycosylation [28, 29]. We first assessed LGALS3BP protein levels in tumor tissues and paired normal tissues using WB. LGALS3BP expression was significantly higher in tumor tissues compared to normal tissues (Figure 5A and 5B, and Figure S5A). Unexpectedly, LGALS3BP levels increased markedly following *ST6GAL1* overexpression in SW48 cells (Figure S5B). Consistent with these findings, IFC analysis showed elevated LGALS3BP expression in the SW48-OE group, with co-localization observed alongside SNA (Figure S5C). Next, we knocked down *LGALS3BP* expression in *ST6GAL1*-overexpressing SW48 cells. Both WB and IFC confirmed a significant reduction in LGALS3BP levels after knockdown (Figure 5C and 5D). Notably, the ability of *ST6GAL1* to promote tumor cell proliferation, migration, and colony formation was significantly impaired following *LGALS3BP* knockdown in SW48-OE cells (Figure 5E–5G and Figure S5D). Moreover, *LGALS3BP* knockdown reversed the chemoresistance of SW48-OE cells to 5-FU treatment (Figure 5H). In conclusion, our findings demonstrate that LGALS3BP, a substrate of ST6GAL1, plays a critical role in mediating the protumor functions of ST6GAL1 in CRC. Its knockdown effectively blocks these tumor-promoting effects.

### Removal of sialylation blocks the effects of ST6GAL1 on CRC cells

**Figure 6.**
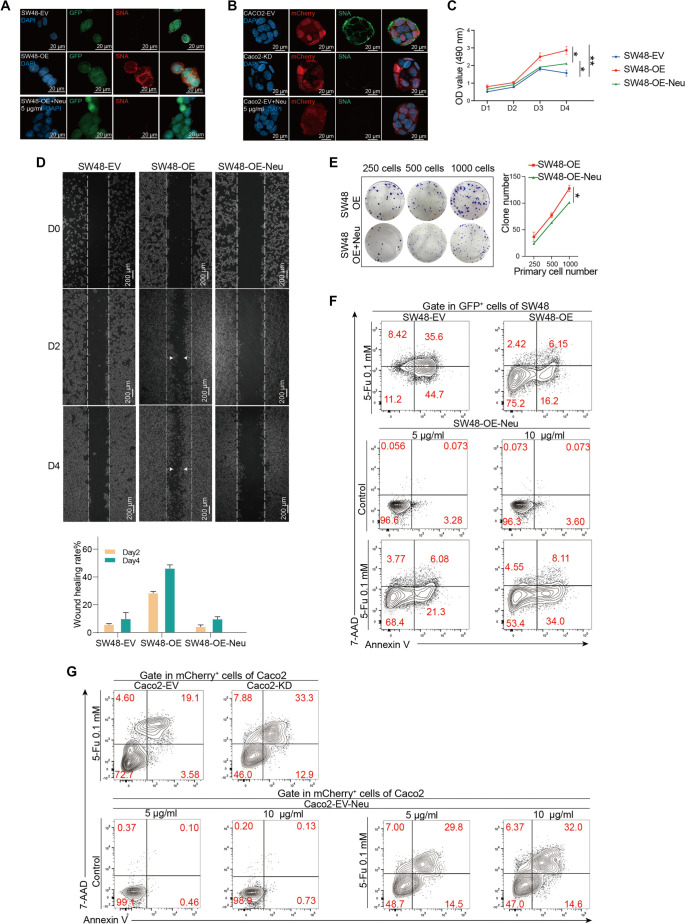
**Desialylation blocks CRC cell proliferation, migration, and chemoresistance.** SW48-EV or Caco2-OE cells were treated with α-(2-3, 6, 8, 9) neuraminidase (NA) for 24 h at a concentration of 5 µg/mL. (A and B) IFC staining of SNA (red) in SW48-EV (GFP, green) and SW48-OE (GFP, green) and SW48-OE (GFP, green) cell lines treated with NA (5 µg/mL) for 24 h. Nuclei were counterstained with DAPI (blue) (scale bars, 20 µm); (B) IFC staining of SNA (green) in Caco2-EV (mCherry, red) and Caco2-KD (mCherry, red) cell lines and Caco2-EV (mCherry, red) cell lines treated with NA (5 µg/mL) for 24 h. Nuclei were counterstained with DAPI (blue) (scale bars, 20 µm); (C) MTT assay results in SW48-OE cells; (D) Wound healing assay of SW48-OE cells on days 0, 2, 4 (scale bars, 200 µm) and corresponding statistical results; (E) Colony formation assay in SW48-OE cells; (F and G) SW48-OE (F) or Caco2-EV (G) cells were treated with or without 5-Fu combined with NA (5 µg/mL and 10 µg/mL) for 48 h, after which Annexin V and 7-AAD staining was detected via flow cytometry. *P < 0.05, **P < 0.01, by Student’s *t*-test (C and E). The data are presented as the mean ± SD of three independent experiments. SNA: Sambucus nigra lectin; MTT: Methyl thiazolyl tetrazolium; IFC: Immunofluorescence.

To confirm the effects of ST6GAL1-induced sialylation on CRC cells, we treated SW48-OE and Caco2-EV cells with sialidase (NA), which significantly decreased sialylation in both cell lines, as evidenced by reduced SNA lectin and IFC staining (Figure 6A and 6B, and Figure S6A). Following NA treatment, the ability of ST6GAL1 to promote tumor cell proliferation, migration, and colony formation was significantly inhibited (Figure 6C–6E). Additionally, the resistance conferred by *ST6GAL1* overexpression against 5-FU chemotherapy was reversed by desialylation with NA (Figure 6F). Furthermore, NA treatment markedly reduced tumor cell proliferation, migration, and colony formation in Caco2-EV cells (Figure S6B–S6D). Similarly, desialylation decreased chemoresistance in Caco2-EV cells (Figure 6G). These findings indicate that the protumor functions of ST6GAL1 are primarily mediated through substrate sialylation.

## Discussion

In addition to increasing protein stability and diversity in normal tissues, sialylation plays a critical role in various biochemical and physiological activities in eukaryotic cells. However, aberrant sialylation and elevated *ST6GAL1* expression are increasingly recognized as features of CRC and many other cancer types [18]. Several sialylated proteins, such as PSA, CA125, and THYROGLOBULIN, are already used clinically to monitor cancer progression and recurrence [6]. Despite this, the substrates of sialylation and the functions of sialyltransferases in cancer cells remain largely unexplored as potential biomarkers or therapeutic targets.

In this study, we confirmed that both sialylation and *ST6GAL1* are upregulated in CRC. *In vitro* data revealed that increased *ST6GAL1* expression promoted CRC cell proliferation, migration, and chemoresistance, findings further supported by transcriptomic and glycosylation modification sequencing data. Notably, the removal of sialylation completely blocked the protumor activity of ST6GAL1, underscoring the importance of sialylation in cancer cells.

To investigate the global protumor activity of ST6GAL1, we performed transcriptomic sequencing following *ST6GAL1* overexpression and observed upregulation of numerous genes related to EMT, proliferation, migration, and metastasis. Nidogen 1 (NID1), a component of the extracellular matrix and basement membrane, facilitates interactions with laminins, collagens, and proteoglycans, thereby activating EMT and regulating cancer cell polarization, migration, and invasion [30, 31]. Our data revealed higher *NID1* expression in cell lines with elevated *ST6GAL1* levels. Specifically, *ST6GAL1* overexpression in SW48 cells significantly enhanced *NID1* expression, while *ST6GAL1* knockdown in Caco2 cells led to its marked reduction.

Similarly, the sex-determining region Y-box protein 5–Twist family BHLH transcription factor 1 pathway, a master regulator of EMT in prostate [32] and gastric cancers [33], was significantly upregulated in *ST6GAL1*-overexpressing SW48 cells. Cyclin-related genes, such as *CDKN2A*, were also upregulated in these cells, while genes like *CDC45*, *CCNB2*, *CDKN3*, and *CCNA2* were downregulated in *ST6GAL1*-knockdown Caco2 cells. Additionally, tumor adhesion and metastasis-promoting molecules, including *ITGB4* and *ITGB8* [34, 35], whereas *ITGA1* and *ITGA2* were downregulated in *ST6GAL1*-knockdown cells. Growth factor-related transcription factors, such as the *MYC* proto-oncogene—an important regulator of tumor development and progression [36]—were significantly downregulated in *ST6GAL1*-knockdown Caco2 cells. These widespread changes in gene expression align with the protumor activity of ST6GAL1.

As an important sialyltransferase, ST6GAL1’s protumor activity likely depends on its substrate sialylation. To further investigate, we conducted N-glycoproteome analysis following *ST6GAL1* overexpression, identifying numerous sialylated substrates associated with protumor pathways, such as wound healing and proliferation. For example, sialylation of ITGB1 has been linked to increased chemoresistance in tumor cells [10, 11, 37]. Moreover, we identified novel substrates like LGALS3BP [38], ITGB8 [39], CD36 [40], and ECE1 [41], which may contribute to malignant tumor progression. LGALS3BP, part of the β-galactoside-binding protein family involved in cell–cell and cell–matrix interactions, is known to be modified by terminal sialic acids and fucose [42–44]. High LGALS3BP expression in various tumors correlates with poor clinical outcomes, and its protumor activity primarily depends on its secreted, N-glycosylated form [28, 29].

Our data revealed that LGALS3BP is a direct sialylation substrate of ST6GAL1, potentially explaining part of ST6GAL1’s protumor activity. Importantly, the knockdown of *LGALS3BP* significantly inhibited the proliferation, invasion, and chemoresistance of *ST6GAL1*-overexpressing cells. Moreover, *ST6GAL1* overexpression increased the migration of SW48 cells, further highlighting the role of LGALS3BP sialylation in regulating tumor cell migration. These findings suggest that future studies should focus on elucidating the functions and mechanisms of sialylation and sialylated molecules, which may reveal new therapeutic targets.

In summary, ST6GAL1 and its sialylated substrates are garnering increasing attention for their clinical potential. However, the mechanisms underlying the malignant behaviors of tumors are complex and multifaceted. While sialylation is clearly associated with malignant transformation, other sialyltransferases, such as ST6GAL2 and ST3GAL4, also regulate sialylation, and their roles cannot be dismissed. Our omics results indicated that certain molecules, though not directly sialylated, are affected by *ST6GAL1* overexpression, suggesting that sialic acid modification by ST6GAL1 influences tumor cell function.

Together with our *in vitro* results, we demonstrated that *ST6GAL1* upregulation is associated with enhanced malignant characteristics, such as tumor proliferation and migration, indicating that ST6GAL1 may contribute to increased tumor aggressiveness. Despite the promising *in vitro* and omics findings, further *in vivo* studies are necessary to validate *ST6GAL1*, LGALS3BP, and sialylation as clinical targets for CRC treatment. Ultimately, exploring the roles of other sialic acid family members in tumor biology remains an important area for future research.

## Conclusion

This study identifies a novel mechanism by which ST6GAL1 promotes CRC progression. It demonstrates that ST6GAL1 facilitates CRC development through the sialylation of LGALS3BP and the upregulation of protumor genes, contributing to tumorigenesis and chemoresistance. These findings offer a valuable perspective and suggest new directions for future CRC treatments.

## Supplemental data

Supplemental data are available at the following link: https://www.bjbms.org/ojs/index.php/bjbms/article/view/11663/3740.

## Data Availability

Data will be made available on reasonable request.
